# Wettability and morphology of proboscises interweave with hawkmoth evolutionary history

**DOI:** 10.1242/jeb.245699

**Published:** 2023-10-11

**Authors:** Alexandre V. Palaoro, Akshata R. Gole, Yueming Sun, Adam Puchalski, Charles E. Beard, Peter H. Adler, Konstantin G. Kornev

**Affiliations:** ^1^ Department of Materials Science and Engineering, Clemson University, Clemson, SC 29634, USA; ^2^ Department of Plant and Environmental Sciences, Clemson University, Clemson, SC 29634, USA; ^3^ Department of Biological Sciences, Clemson University, Clemson, SC 29634, USA

**Keywords:** Contact angle, Menisci, Fluid dynamics, Lepidoptera, Sphingidae

## Abstract

Hovering hawkmoths expend significant energy while feeding, which should select for greater feeding efficiency. Although increased feeding efficiency has been implicitly assumed, it has never been assessed. We hypothesized that hawkmoths have proboscises specialized for gathering nectar passively. Using contact angle and capillary pressure to evaluate capillary action of the proboscis, we conducted a comparative analysis of wetting and absorption properties for 13 species of hawkmoths. We showed that all 13 species have a hydrophilic proboscis. In contradistinction, the proboscises of all other tested lepidopteran species have a wetting dichotomy with only the distal ∼10% hydrophilic. Longer proboscises are more wettable, suggesting that species of hawkmoths with long proboscises are more efficient at acquiring nectar by the proboscis surface than are species with shorter proboscises. All hawkmoth species also show strong capillary pressure, which, together with the feeding behaviors we observed, ensures that nectar will be delivered to the food canal efficiently. The patterns we found suggest that different subfamilies of hawkmoths use different feeding strategies. Our comparative approach reveals that hawkmoths are unique among Lepidoptera and highlights the importance of considering the physical characteristics of the proboscis to understand the evolution and diversification of hawkmoths.

## Teaser

**Highlighted Article:** The long proboscises of hawkmoths are entirely hydrophilic, a pivotal discovery for understanding the evolution of hawkmoths, setting them apart from other Lepidoptera. Hydrophilic proboscises facilitate nectar collection as a film that is spontaneously pulled into the food canal by strong capillary action.

## INTRODUCTION

Ever since Charles Darwin predicted that an orchid with an extraordinarily long nectar spur might be pollinated by a moth with an equally long proboscis (Arditti et al., 2012; Darwin, 1877), and Alfred Russel Wallace (Arditti et al., 2012; Wallace, 1867) subsequently suggested that hawkmoths (Lepidoptera, Sphingidae) were likely candidates, hawkmoths have been popular models for understanding ecomorphological consequences in biology (Krenn and Kristensen, 2000; Kristensen, 1984; Kristensen et al., 2014) and bio-inspired engineering applications (Dudley, 2002; Stöckl and Kelber, 2019; Zhang et al., 2019). The family Sphingidae comprises more than 1460 species (Goldstein, 2017; Lees and Zilli, 2019). An understanding of the evolutionary diversification of hawkmoths and the morphology of their feeding organs (Bauder et al., 2015; Ferry and Higham, 2022; Wainwright, 2007; Wainwright and Reilly, 1994; Wainwright et al., 2005) requires an understanding of the links among proboscis structure, function and performance (Krenn and Aspöck, 2012; Lauder, 2003; Muñoz, 2019; Russell, 1916; Wainwright, 2007).

Hawkmoths are well known for foraging on nectar from flowers, often while hovering (Arditti et al., 2012; Houlihan et al., 2019; Johnson et al., 2017; Lees and Zilli, 2019; Miller, 1997; Raguso and Willis, 2003; Wasserthal, 1997). Hovering is one of the most metabolically expensive forms of locomotion (Haverkamp et al., 2016; Kammer and Heinrich, 1978). Therefore, hawkmoths need to offset the energy expended during hovering. Any behavioral or morphological trait that increases feeding performance should be under strong selection pressure (Haverkamp et al., 2016). Two primary biomechanical phenomena can increase proboscis performance: (1) passive collection of fluid into the proboscis through physical forces (e.g. capillary and wetting forces), and (2) active movement of collected fluid through the food canal, mediated by powerful muscles (i.e. the sucking pump) in the head cavity (Kornev and Adler, 2019). Previous studies on the butterfly proboscis show that wetting and morphological properties, which are responsible for passive fluid collection, significantly affect the rate of liquid uptake (Kwauk et al., 2014; Lehnert et al., 2013; Tsai et al., 2014), making these properties possible targets of selection. Rapid drinking could also diminish predation rates by decreasing the time spent at the same food patch (Arditti et al., 2012; Josens and Farina, 2001; Martins and Johnson, 2013; Pauw et al., 2009; Wasserthal, 1997). In this paper, we studied the passive mechanisms of fluid uptake by hawkmoth proboscises.

**Fig. 1.**
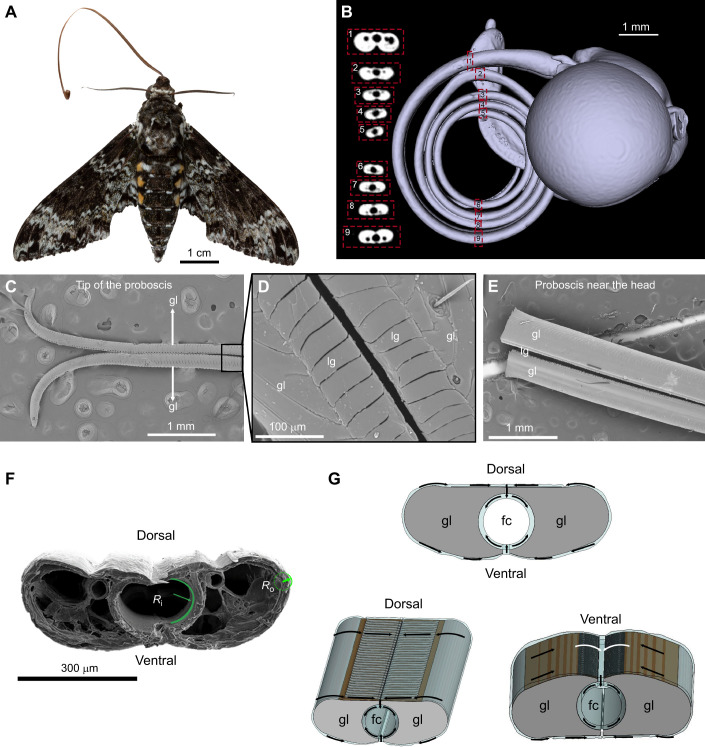
**The hawkmoth proboscis.** (A) *Manduca rustica* (dorsal view) with partially uncoiled proboscis. (B) Micro-computed tomography (micro-CT) scan of the head (lateral view) of *M. rustica*, with a sequence of proboscis cross-sections on the left. (C) Scanning electron microscopy (SEM) images of the tip of the proboscis of *M. rustica*, with galeae (gl) ‘unzipped’ during preparation for SEM. (D) The boxed region in C shown at higher magnification, where legulae (lg), the bar-like cuticular projections, form the dorsal legular band of the proboscis. This dorsal legular band consists of the two rows of legulae, one row per galea. The legulae are separated from one another by liquid-permeable gaps (dark lines between adjacent legulae). (E) Two separated galeae of the proboscis of *M. rustica*. This image highlights the structure of the proboscis near the head. Given the proximity to the head, the galeae were cut to be able to position the proboscis for imaging. The legulae in this region are shorter than those near the proboscis tip shown in C and D. (F) SEM image of the cross-section of the proboscis of *Manduca sexta*. The C-shaped faces of each galea form a food canal. The galeae are linked by dorsal and ventral legulae. The radius of curvature of the food canal (*R*_i_) and the smallest radius of curvature of the galea (*R*_o_) are shown. (G) Schematic diagram of the proboscis and fluid flow from the external film (blue) of the proboscis surface into the food canal (fc). We show three views of the proboscis, a cross-section, one view of the dorsal side and another view of the ventral side. The arrows within the film indicate the direction of liquid flow (note, in nature, this film is much thinner, barely visible). A darker layer was added beneath the legulae to highlight interlegular spaces created between adjacent legulae.

Contrary to popular belief, the lepidopteran proboscis is not simply a drinking straw (Monaenkova et al., 2012); that is, it is not an open-ended tube with a solid liquid-impermeable shell where the fluid could only flow under the applied pressure from the sucking pump, as some earlier models suggest (Kingsolver and Daniel, 1979). The proboscis is composed of two C-shaped tubes, the galeae (Fig. 1A–D), that are united to form the food canal after the insect emerges from the pupa (Sande et al., 2021; Zhang et al., 2018b). The legulae (Fig. 1D), a series of cuticular projections, run the entire length of the proboscis on both the dorsal and ventral sides, forming dorsal and ventral legular bands, which are permeable to fluids (Fig. 1E,F). Adjacent legulae are separated from one another, creating a sieve-like legular band along the axis of the proboscis, which allows fluids to enter the food canal (Kornev and Adler, 2019; Salamatin et al., 2021).

For butterflies, only about 10% of the external surface of the proboscis near the tip is hydrophilic (Lehnert et al., 2013, 2016), which means that aqueous food can wet and freely flow over the proboscis surface in the tip region. The legulae are also hydrophilic throughout the entire length of the proboscis (Lehnert et al., 2013; Zhang et al., 2018b); thus, nectar or any other liquid would preferentially enter the food canal through the legular bands (Fig. 1G) (Salamatin et al., 2021; Zhang et al., 2018a). Although liquid also could enter the food canal through the permeable bands along the length of the butterfly proboscis, it hardly does so because liquids bead up on the hydrophobic surface. The contact lines of these droplets get pinned to the surface and the droplets barely move toward the hydrophilic legular bands. Therefore, the greater the hydrophilic area of the proboscis over which the liquid can move, the easier it is for the insect to collect the liquid (Kornev and Adler, 2019). Here, we asked whether hawkmoth proboscises follow the trend of butterfly proboscises with a hydrophobic–hydrophilic dichotomy. To do so, we introduced two quantitative metrics: the contact angle and capillary pressure.

The metric for proboscis wettability is the contact angle (Denny, 1995) that the water meniscus makes with the proboscis. The smaller the angle, the more hydrophilic the proboscis. Given that the proboscis has a lengthwise permeable band, having nectar stuck to the entire length increases nectar uptake rate (Kwauk et al., 2014; Lehnert et al., 2013; Tsai et al., 2014).

**Fig. 2.**
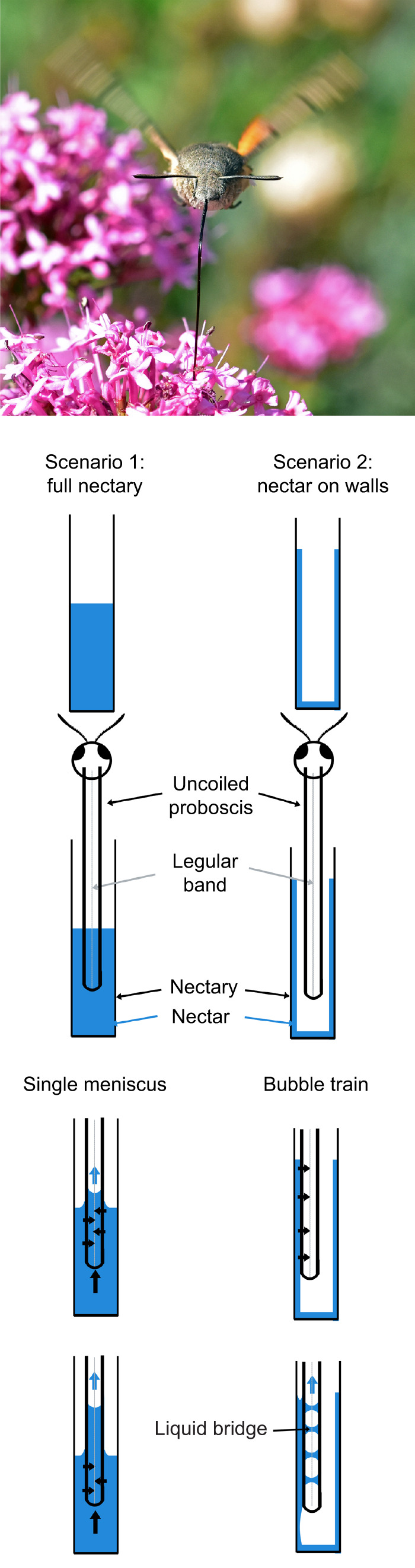
**Possible mechanisms of fluid uptake by a hovering hawkmoth.** (A) When a hawkmoth (*Macroglossum stellatarum* in the image, courtesy of Andrew Dejardin) approaches the flower and dips its proboscis into a nectary, it can encounter two scenarios of nectar deposition, which can influence how fluid enters the proboscis through the dorsal and ventral legular bands. On the left (from top to bottom), the nectary is full of nectar. The hawkmoth uncoils its proboscis and dips it into the nectar. The nectar then moves to the food canal, forming a single meniscus. The lower capillary pressure (black arrows) at the meniscus pulls the nectar through the legular bands (gray line), and upward, filling the food canal. On the right (from top to bottom), the nectar forms a film at the wall of the nectary. When the proboscis touches the film, nectar wets the proboscis and spreads toward the legular bands and into the food canal, where it forms a film with a lower capillary pressure. When the amount of nectar is not sufficient to form a continuous column of nectar in the food canal, the internal film collapses due to Plateau–Rayleigh instability to form a bubble train with liquid bridges separating the air bubbles. The bubble train is then swallowed by the hawkmoth.

The shape of the proboscis aids in moving liquid to the legular bands by employing capillary action (Figs 1F,G and 2) (Salamatin et al., 2021; Zhang et al., 2018a). The metric for this action is the dimensionless capillary pressure (Vogel, 1996), defined by the Laplace law of capillarity. The Laplace law states that the curved liquid–air interface with surface tension γ generates a pressure differential Δ*p* between the liquid under the interface and the air. When the insect pulls its proboscis from a liquid, a fluid film is deposited on the proboscis surface and this film can penetrate the legular bands to wet the food canal of radius *R*_i_ (Fig. 1G). Based on the capillary pressure concept (see Materials and Methods, ‘Formulation of the pressure amplification metric for a straight proboscis’) we introduced a metric (Δ*pR*_i_)/γ similar to the mechanical advantage of levers (Muñoz et al., 2018; Wainwright, 2007). The larger the ratio (Δ*pR*_i_)/γ, the stronger the capillary pull of a liquid film on the proboscis into the food canal. The limiting value (Δ*pR*_i_)/γ=1 is reached when the external radius *R*_o_ of the proboscis becomes much greater than the radius of the food canal, *R*_o_/*R*_i_<<1. In this case, the capillary pressure squeezing the film collected on the proboscis surface toward the legular bands is weaker than the capillary pull from the film in the food canal. Therefore, by tracking the ratio *R*_o_/*R*_i_, one could evaluate which fluid uptake mechanism is dominant, either capillary pull from the food canal (i.e. *R*_o_/*R*_i_>>1) or capillary push from the external film (i.e. *R*_o_/*R*_i_<1).

Thus, the performance metrics characterizing the ability of hawkmoths to take up fluid are the contact angle and the ratio *R*_o_/*R*_i_. Using these parameters, we brought together the physical properties of the proboscis and its morphology to see hawkmoth diversification through the lens of biomechanics. The fluid mechanics of nectar flow inside the food canal is beyond the scope of this paper, but the schematics of two possible scenarios of fluid uptake are given in Fig. 2 and Results.

We investigated the wetting and morphological properties of hawkmoth proboscises and set up quantitative metrics, allowing a comparative evolutionary analysis of the performance of hawkmoths, with respect to fluid uptake.

We evaluated the wettability of 13 species of hawkmoths by measuring the contact angle between water and the proboscis surface, using capillary-rise experiments. We also measured the radius of curvature of the galea and the food canal through analysis of micro-computed tomography (micro-CT) scans. We show that the hawkmoth is unique among Lepidoptera in having a proboscis that is entirely hydrophilic, which allows hawkmoths to collect and deliver fluid to the food canal along its entire length. Our results suggest that wettability is interwoven with the evolutionary history of hawkmoths: the average contact angle varies across hawkmoth species, with the subfamily Sphinginae having smaller contact angles compared with those of the subfamily Macroglossinae. No other lepidopteran lineages that we have analyzed have similar wetting properties, suggesting that an entirely hydrophilic proboscis is unique to hawkmoths.

## MATERIALS AND METHODS

### Studied species

We used an aerial hand net to collect moths in Clemson, SC, USA, during the summers of 2021 and 2022. We observed more than 100 hawkmoths feeding from flowers and sampled a subset of 63 individuals of 13 species, including one species, *Darapsa myron*, attracted to ultraviolet (UV) light (Table 1; Table S1). We also used UV light to collect 6 moths from a lineage close to the Sphingidae, the superfamily Noctuoidea (family Erebidae): *Catocala coccinata* Grote 1872; *Catocala epione* (Drury 1773), *Catocala ultronia* (Hübner 1823), *Catocala neogama* (J. E. Smith 1797) and *Catocala robinsonii* Grote 1872. We obtained zebra longwing butterfly pupae, *Heliconius charithonia* (L.), from Shady Oak Butterfly Farm (Brooker, FL, USA) and held them at 30°C, humidity of 60% or higher, and a 16 h:8 h light:dark cycle until emergence. After collection or emergence from pupae, we returned all lepidopterans to the laboratory and measured their proboscis and forewing length as a prelude to wettability measurements.

**Table 1.**
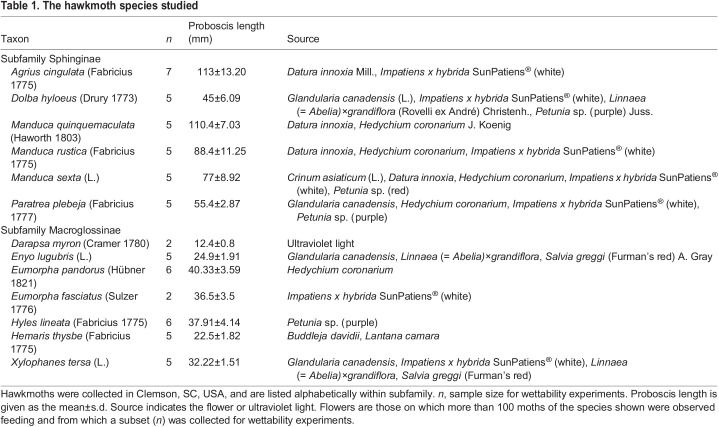
The hawkmoth species studied

### Experiment design

**Fig. 3.**
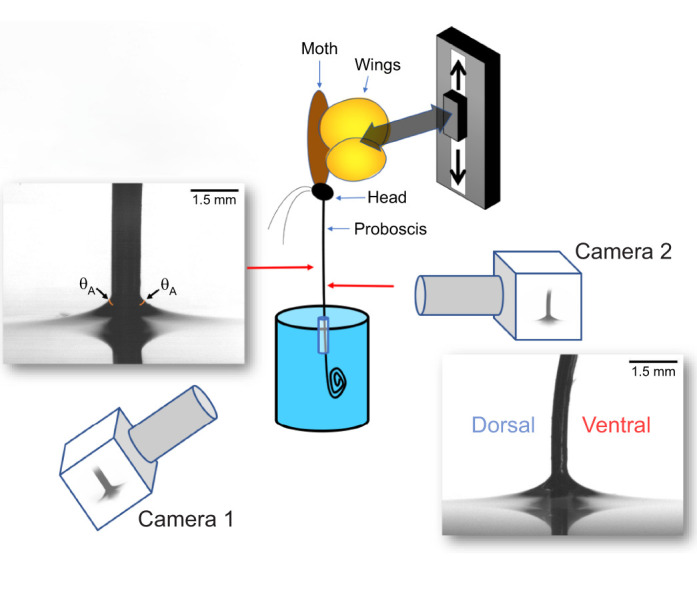
**Schematic diagram of the experiment setup for measuring the contact angle of a proboscis.** The live moth (brown and yellow ellipses) is accommodated on a programmable linear stage. The proboscis is then uncoiled into a beaker filled to the brim (blue rod) with a small straw to keep the proboscis straightened. The insect is moved down to evaluate the advancing contact angle (θ_A_), measured here as the angle the water makes when in contact with the dorsal side of the proboscis. The contact angle is shown in the image of the dorsal side of the proboscis, on both right and left sides. Camera 1 faces the dorsal side of the proboscis to record contact angle formation; camera 2 faces the lateral side of the proboscis to ensure the proboscis remains vertical during movement. Examples of acquired images are given.

The wetting properties of the proboscis were characterized by the contact angle (Denny, 1995) that the water meniscus formed with the sides of the galeae. When a meniscus forms contact angles <90 deg, the surface is hydrophilic. Conversely, when a water meniscus dimples down, forming contact angles >90 deg, the surface is hydrophobic. To model how nectar interacts with the proboscis when a moth dips it into a nectary, we measured the advancing contact angle in deionized (DI) water (Denny, 1995) (θ_A_; Fig. 3). We highlight that the clear division between hydrophilic and hydrophobic at 90 deg angles occurs only when using DI water. Adding substances that alter the surface tension of the water, such as sugar, would change that demarcation. Menisci were not visible on the sides of the butterfly and *Catocala* proboscises: the water surface formed a dimple, hiding the contact line where the water met the proboscis surface. Additionally, we have already shown that only ∼10% of the tip of the butterfly proboscis is hydrophilic (Kornev and Adler, 2019; Lehnert et al., 2013). Thus, we do not report the advancing contact angles for the butterfly or *Catocala*.

To decrease artifacts caused by movements of the moths, we refrigerated live individuals for 5–10 min. The wings, abdomen and thorax of each individual were wrapped in a paper towel and taped at one end to further constrain movement. To hold the individual in place, the taped end was fixed with a wooden clip onto a linear stage (pusher block of an Era Ne-300 digital programmable syringe pump). We then straightened the proboscis, using an insect pin, and guided it through a plastic tube (diameter 3.3 mm) centrally aligned in the beaker beneath the water's surface. An FLIR camera (camera 1 in Fig. 3) with a microscope lens (Navitar 1-60135) and a Halogen light source (F0–150, Fiberoptics Technology, Inc.) were used to record the formation of dynamic contact angles on the lateral sides of the proboscis. The imaging system (camera 2 in Fig. 3) of a Kruss Drop Shape Analyzer was used to observe the dorsal and ventral sides and ensure that the proboscis was vertical during the movement.

To obtain videos showing meniscus formation on the proboscis, the tube was held beneath the water surface. The initial position of the insect was adjusted near the water level, and the distance between the head and water level was marked as a reference, *d*_ref_. The proboscis was pulled upward to uncoil it. In this mode, the receding contact angle was measured. The linear stage was then set to move the insect downward at 0.7 mm s^−1^ to its initial position where the head of the insect was at the reference distance (*d*_ref_) from the water surface. During this process, the advancing contact angle was measured. The time interval between these runs was set at 10 min, so the water film on the proboscis had time to evaporate and the advancing contact angle could be measured. The validity of this protocol was previously confirmed using painted lady butterflies (*Vanessa cardui* L.; Kornev and Adler, 2019; Lehnert et al., 2013). All videos were recorded at an average of 13.45 frames s^−1^ to show the changing meniscus profile on the left and right sides of the proboscis at different positions. The dorsal and ventral legular bands are hydrophilic even in butterflies (Kwauk et al., 2014; Lehnert et al., 2013; Monaenkova et al., 2012), and the same hydrophilicity was observed for the hawkmoth legular bands.

### Measuring the contact angle

We measured the contact angles using a custom LabVIEW code and Fiji-ImageJ (Schindelin et al., 2012; Schneider et al., 2012). We used two methods to ensure the reliability and robustness of our measures (Supplementary Materials and Methods, Figs S1–S3). The advancing and receding contact angles were measured every 2–3% of the proboscis length for each individual. The measurements covered 65–80% of the proboscis length, depending on the length of each proboscis. Regions near the tip were usually not probed because the proboscis would slip from the hold and coil back at those regions. We calculated the mean and standard deviation of the advancing and receding contact angles forming on the hydrophilic part of the galeal sides. The contact angle hysteresis is given by the difference between the advancing contact angle and the receding contact angle (θ_A_−θ_R_) (Supplementary Materials and Methods). The greater the hysteresis, the harder it is to move the drop over the surface.

The following three metrics were introduced from the advancing contact angle measurements. (i) To calculate the average contact angle, we divided the proboscis into sections of 10% of its length divided by the entire proboscis length. We then averaged the contact angles of all individuals of a single species within that section and repeated this for all sections of the proboscis, yielding a mean advancing contact angle profile of the proboscis for each species. (ii) To obtain the overall advancing contact angle, we calculated the mean contact angle by averaging the contact angles along the proboscis of each individual. To obtain the mean contact angle for each species, we averaged the mean contact angles of all individuals of each species. The standard deviation, in this case, was the standard deviation of the averages. Because all measurements closest to the head (normalized distance 0–0.1, or the 10% closest to the head) showed a sharp increase in contact angle (resulting from hydrophobic scales on the head), we did not use these values to calculate the overall mean contact angle. (iii) To calculate the rate of change in contact angle along the proboscis, we fitted a linear regression between the contact angle and section of the proboscis. We then used the slope of this relationship as the rate of change in contact angle along the proboscis. We discarded the measurement closest to the head because of the sharp increase in contact angle caused by the scales on the head.

### Micro-CT analysis of proboscis cross-sections

Hawkmoths were put in an ultra-low freezer at −76°C the day after collection or after they were used in wetting experiments. The frozen hawkmoths were shipped on ice within 2 days to North Dakota State University Electron Microscopy Core Lab in Fargo, ND, USA, for micro-CT. The scanning was conducted within 2 days of receipt of the specimens. Each sample was placed at the top of a Kapton 40 tube with the head protruding. A GE Phoenix v|tome| x s X-ray micro-CT equipped with a 180 kV high-power nanofocus X-ray tube xs|180nf and a high-contrast GE DXR250RT flat panel detector collected the micro-CT images. A molybdenum target was used to acquire 1000 projections of the sample at a voltage of 60 kV and a current of 240 μA. Detector timing was 1500 ms and total acquisition time was 1 h 40 min. Sample magnification was 55.22× with a voxel size of 2.4 μm. The acquired images were reconstructed into a volume dataset using GE datos|x 3D-computer tomography software version 2.2.

### Formulation of the pressure amplification metric for a straight proboscis

In the food canal modeled as a circular cylinder of radius *R*_i_, the Laplace law for a very thin liquid film with thickness *h* much smaller than the food canal radius, *h*<<*R*_i_, gives the capillary pressure 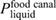
−*P*_air_=−γ/*R*_i_, where γ is the surface tension of the liquid. In contrast, the same film at the outer surface of the galeae, considered as a straight complexly shaped cylinder, has positive capillary pressure. This capillary pressure changes along the galeal cross-sectional profile. At the position where the radius of curvature *R*_o_ is smallest, we have 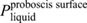
−*P*_air_=γ/*R*_o_ (Fig. 1F). Thus, the pressure in the liquid film inside the food canal is less than atmospheric pressure, but the pressure in the liquid film on the proboscis surface is always greater than atmospheric pressure.

We set up a metric similar to the mechanical advantage for levers to measure the pressure amplification achieved by changing the proboscis morphology: 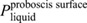
−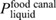
=Δ*p*=γ(1/*R*_o_+1/*R*_i_). To calculate the pressure amplification factor, this formula is rewritten in dimensionless form as:
(1)

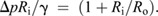


### Curvature and pressure differential calculations

To measure the curvatures of the food canal and galea cross-sections, we used a cross-sectional image of the proboscis coil from the micro-CT scan (i.e. one slice). The cross-sectional image contained several sections of the proboscis due to its coiling (Fig. 1), which allowed us to measure the radius of curvature of several parts of the proboscis and obtain an average radius of curvature for each individual. To measure the radius of curvature, a custom macro for Fiji-ImageJ (Schindelin et al., 2012; Schneider et al., 2012) was written (Supplementary Materials and Methods). The C-faced part of the galea was fitted with a circle. The radius of the best-fit circle was assigned as *R*_i_. To find *R*_o_, we first visually determined the location of the most curved part of the external surface of the galea with the smallest radius of curvature. In some sections of the proboscis, the galea was uniformly curved in a circular arc but, as in Fig. 1F, the outermost part of the galea is not always circular. Thus, we looked for the most curved part and fitted it with a circle. The radius of the best-fit circle was assigned as *R*_o_. We repeated the process four times and averaged the circumradii to obtain the radius of curvature. We then performed the same process on the other galea and averaged both sides. The radius of curvature of one individual was an average of the radii of all the sections of the proboscis. Our visual assessment of the most curved part was reliable because the standard deviations are low (Table S1). To calculate the radius of curvature of the food canal, we used Slicer 3D 5.0.3 to reconstruct the volume of the proboscis (Antiga et al., 2008; Fedorov et al., 2012). We manually segmented the food canal as a volume, extracted its center line and calculated the diameter along the center line, using the Vascular Modeling Toolkit (Antiga et al., 2008). Given that the food canal cross-section is circular, the radius of curvature is the radius itself.

With both radii of curvature, we calculated the dimensionless pressure differential using Eqn 1.

### Statistical analyses

We conducted all statistical analyses in R software (http://www.R-project.org/). For the evolutionary relationships of hawkmoths, we used a multigene, time-calibrated molecular phylogeny (Kawahara and Barber, 2015). We pruned the tree for our target species. The only species not present in the tree was *Eumorpha fasciatus*, which we manually added next to its congener (Ponce et al., 2015), *Eumorpha pandorus*, using the *bind.tip()* function in the package *phytools* (Revell, 2012).

Our goal was to test how the capillary pressure, tapering angles (the rate at which the galeal width decreases along the proboscis) and contact angles correlated with each other and with proboscis length while accounting for phylogeny. Our first phylogenetic linear regression used the pressure differential measure (ratio between outer and inner radii of curvature) as a response variable and the average advancing contact angle as our predictor variable. We modelled trait evolution using Brownian motion, Lambda, Kappa and Delta models (Tung Ho and Ané, 2014). We did not use more complex models of trait evolution (e.g. Ornstein–Uhlbeck, Early Burst) because the number of species could favor more complex models as a result of the difficulties of estimating all parameters with samples lower than 30 (Cooper et al., 2016). To distinguish which model best fitted our data, we compared them using Akaike's information criterion (AIC). We chose the model with the lowest AIC as the best fit so long as it had more than 2 units of AIC of difference from the other models (Burnham and Anderson, 2004). After we found the evolutionary model that best fitted our data, we ran a bootstrap with 10,000 replications to calculate the confidence intervals of the estimated parameters. We performed a similar analysis for the tapering angle, using it as the response variable and the advancing contact angle as the predictor variable. We modelled trait evolution similarly.

For the next analyses, we first standardized the length of the proboscis by dividing it by the forewing length. By using a ratio between proboscis length and forewing length, we accounted for body size effects on proboscis length, while also having a dimensionless metric for size. Thus, our test included the average advancing contact angle as our response variable, a ratio between proboscis length and forewing length as our predictor variable, and an error measurement associated with the contact angle. Trait evolution modeling followed a similar method to that described above. In the next analysis, we tested whether the slope of change of the advancing contact angle along the proboscis was correlated with relative proboscis length. To do so, we used a phylogenetic linear regression with the slope of change of the advancing contact angle as a response variable and the ratio between proboscis length and forewing length as our predictor variable. The error measurement and trait evolution were similar to the previous phylogenetic regression. We performed all phylogenetic linear regressions using the package *phylolm* (Tung Ho and Ané, 2014). Lastly, we drew phenograms of the average advancing contact angle and the slope of change, using the function *phenogram()* in the package *phytools* (Revell, 2012).

## RESULTS

### Hawkmoths have a hydrophilic proboscis

**Fig. 4.**
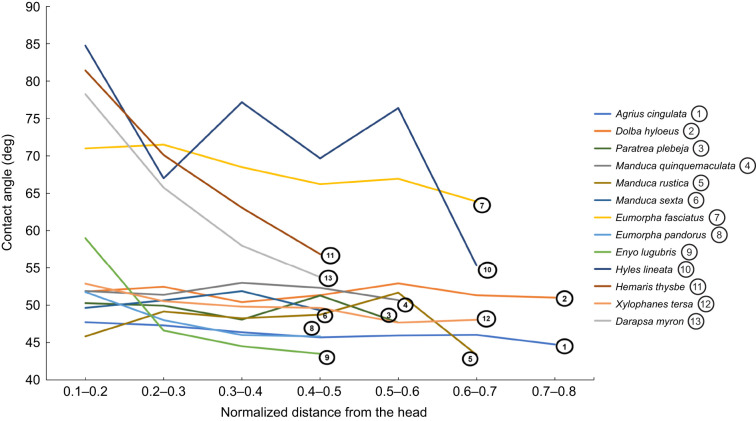
**Average advancing contact angles along the proboscis of 13 species of hawkmoths.** The normalized distance from the head is the ratio obtained from the location on the proboscis where the contact angle was measured divided by the length of the entire proboscis. The contact angle is reported for each 10% interval along the proboscis length. Lower values indicate locations toward the head, whereas larger values (up to 1) indicate areas toward the tip. Species differ in proboscis length, but plotting these results in normalized variables allows us to compare species. Standard deviations were omitted to enhance clarity of the trends (Table S2).

Advancing contact angles of all hawkmoth species were smaller than 90 deg for most or all the length of the proboscis (Fig. 4). For species such as *Paratrea plebeja*, *Manduca rustica*, *Agrius cingulata* and *Manduca sexta*, the proboscis was hydrophilic for the entire length. For species such as *Hemaris thysbe*, *D. myron* and *Hyles lineata*, 80–90% of the proboscis was hydrophilic (i.e. all but 10–20% of the basal length).

### Feeding behaviors of hawkmoths

Hawkmoths fed from a wide diversity of plant species with different floral morphologies (Table 1). Only hawkmoths with the longest proboscises (*A. cingulata*, *Manduca quinquemaculata*, *M. sexta* and *M. rustica*) were observed feeding from flowers with the longest floral tubes (*Datura innoxia*), whereas only species with the shortest proboscises (e.g. *Enyo lugubris*, *H. thysbe* and *Xylophanes tersa*) were observed feeding from flowers with the shortest floral tubes (e.g. *Glandularia canadensis*, *Lantana camara* and *Salvia greggi*) (Table 1). We do not imply that species with long proboscises cannot feed from flowers with short floral tubes; rather, it was not observed in our study, perhaps because preferred flowers were in full bloom.

**Fig. 5.**
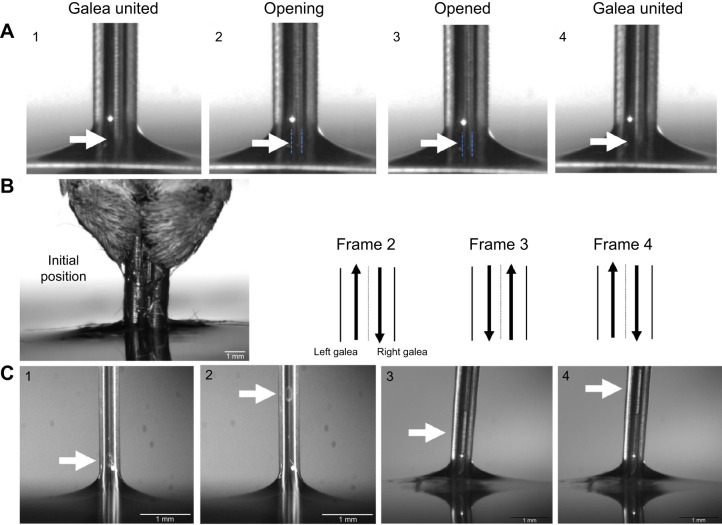
**Movements of the galeae during fluid uptake.** (A) Reversible ‘cracking’ of the dorsal legular band at different locations was observed when the proboscis of *M. rustica* was lowered into water. The ‘crack’ or opening in the dorsal legular band allowed water to rush into the food canal. The crack was closed again in frame 4. Numbers 1–4 show the sequence of events. (B) Anti-parallel sliding of the galeae in *Eumorpha pandorus*. Arrows indicate the direction of the movement of each galea. Movie 2 shows the process. (C) Movement of air bubbles (arrows) can be seen in the proboscis of *M. sexta* (1 and 2) and *Manduca quinquemaculata* (3 and 4), suggesting that the liquid column in the food canal is partitioned as bubble trains.

In the laboratory, we observed three behaviors of the proboscis associated with feeding by hawkmoths with long proboscises. In *M. rustica*, we observed what we called ‘cracking’ of the proboscis (Fig. 5A); that is, a slight opening of the dorsal legular band along the axis of the proboscis. Cracking happened when either a single galea or both galeae twisted with respect to the galea axis. This localized twist slightly ‘unzips’ the band by causing the legulae to split apart and open a space where water rushes in. Cracking occurred at different locations along the proboscis, suggesting that the moth can open and close the legular bands on demand while not entirely separating the galeae. Water rushes into the crack, facilitating filling of the entire food canal.

We observed anti-parallel sliding of the galeae (Fig. 5B; Movie 2) in 10 species (*A. cingulata*, *D. myron*, *Dolba hyloeus*, *E. lugubris*, *E. pandorus*, *H. thysbe*, *H. lineata*, *M. quinquemaculata*, *P. plebeja* and *X. tersa*). Anti-parallel sliding consists of independent movement of the galeae in opposite but parallel directions along the length of the proboscis. The legulae of each galea slide over those of the other galea, changing the width of the interlegular gaps. In this sliding process, the galeae remain united. The only opening of the food canal occurs at the tip for water to rush in or when the galea cracks (Movie 1).

We also observed the bubble-train phenomenon registered with X-ray phase contrast imaging in butterflies (Monaenkova et al., 2012; Fig. 5C; Movie 3), where bubble trains are formed in the food canal to reduce friction of the liquid column, thus reducing the dissipation of muscular energy provided by the sucking pump.

### Proboscis morphology ensures capillary pressure differential

**Fig. 6.**
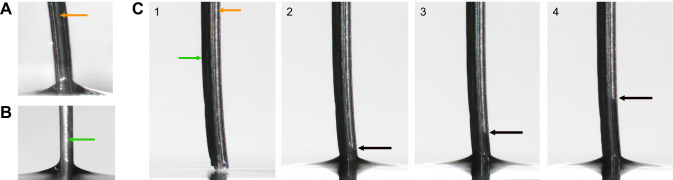
**Capillary action in a single galea.** (A,B) The inside (i.e. the food canal; A, orange arrow) and the outside (lateral side; B, green arrow) of one galea. (C) An image sequence showing the capillary action outside and inside the galea. The camera was positioned at a 45 deg angle that allowed filming of the food canal (orange arrow) and the lateral side of the same galea (green arrow) simultaneously. The first image shows the moment before the galea touches the water, and the sequence shows the water rising up the food canal (black arrow). To perform this experiment, we cut the proboscis of a dead hawkmoth, which reinforces that capillary action is a property of the structure of the proboscis. Movie 4 shows the full process.

The two radii of curvature, *R*_i_ and *R*_o_, provide a useful metric for evaluating capillary action offered not only by the united proboscis but also by a single galea (as defined in Fig. 1). The external part of the galea, having the cross-sectional radius *R*_o_ at its outermost bulged side, tends to squeeze out the liquid film after proboscis withdrawal, while the indented part of the food canal, having radius *R*_i_, tends to attract the liquid (Fig. 6). In this capillary rise experiment, as soon as the galea touches the water surface, water rushes into the food canal while forming a small meniscus outside (Movie 4). The height of the liquid column inside the C-shaped food canal is incomparably greater than the height of the external meniscus, confirming this statement. Far from the meniscus where the galea meets the water surface, the positive capillary pressure *P*_liquid_−*P*_air_≈γ/*R*_o_ pushes the liquid from the external surface of the galea while the negative capillary pressure *P*_air_−*P*_liquid_≈γ/*R*_i_ pulls the liquid into the C-shaped channel of the food canal (Tsai et al., 2014; Zhang et al., 2019). When the galea is withdrawn from the water, the liquid column remains trapped in the C-shaped food canal (Movie 4) but the external surface of the galea is dry.

**Fig. 7.**
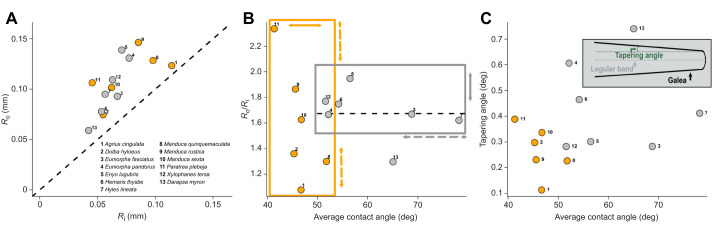
**Proboscis morphology ensures a capillary pressure differential that enables a liquid film to be transported into the food canal.** (A) Ratio between the radius of curvature of the external surface of the galeae, *R*_o_, to the radius of curvature of the food canal, *R*_i_. The dashed line corresponds to *R*_o_/*R*_i_=1. All species have *R*_o_ values above the dashed line, suggesting that the driving force for accumulation of liquid film from the external surface to the food canal is the capillary pressure of the liquid film covering the food canal. The orange circles represent species of the subfamily Sphinginae, and the gray circles represent species of the subfamily Macroglossinae (Table 1). (B) Lack of a functional correlation between *R*_o_/*R*_i_ and the average contact angle. This set of data points select Sphinginae as having advancing contact angles less than ∼55 deg with a broad range of *R*_o_/*R*_i_ ratios. Macroglossinae reside in a band 1.6<*R*_o_/*R*_i_<1.9 but with advancing contact angles greater than 55 deg. The dashed line represents the average *R*_o_/*R*_i_ of the subfamily. Dashed arrows show the axis of largest variation, while solid arrows show the axis of smallest variation. The colors of the arrows match the subfamily (Sphinginae in orange, Macroglossinae in gray). (C) Tapering angle of the proboscis is also not correlated with the advancing contact angle.

In all species analyzed, the radius *R*_o_ of curvature of the galea was always greater than the radius *R*_i_ of the food canal (Fig. 7A). Hence, the driving force for flow of a liquid film from the external surface of the galea to the food canal is the capillary pressure generated at the film surface of the food canal. This pressure is sufficient to direct the liquid to the dorsal and ventral legular bands where it can be absorbed by the food canal. The ratio *R*_o_/*R*_i_, however, was not correlated with the advancing contact angle (slope: −0.004 [95% confidence interval, CI: −0.02, 0.011]; *t*=−0.53; *P*=0.301; adjusted *R*^2^=−0.06; Fig. 7B; Table S3a). The tapering angle also was not correlated with the advancing contact angle (slope: 0.005 [95% CI: −0.002, 0.013]; *t*=1.23; *P*=0.24; adjusted *R*^2^=0.04; Fig. 7C; Table S3b).

Two selection pressures are evident in the evolution of hawkmoth proboscises (Fig. 7B,C). Macroglossinae evolved with greater scatter of the advancing contact angle and tapering angle, but a narrower range for *R*_o_/*R*_i_. Sphinginae, in contrast, evolved with smaller advancing contact angles and tapering angles, but with *R*_o_/*R*_i_ varying within a wide range.

### Average advancing contact angle and contact angle gradient decrease as the proboscis becomes longer

**Fig. 8.**
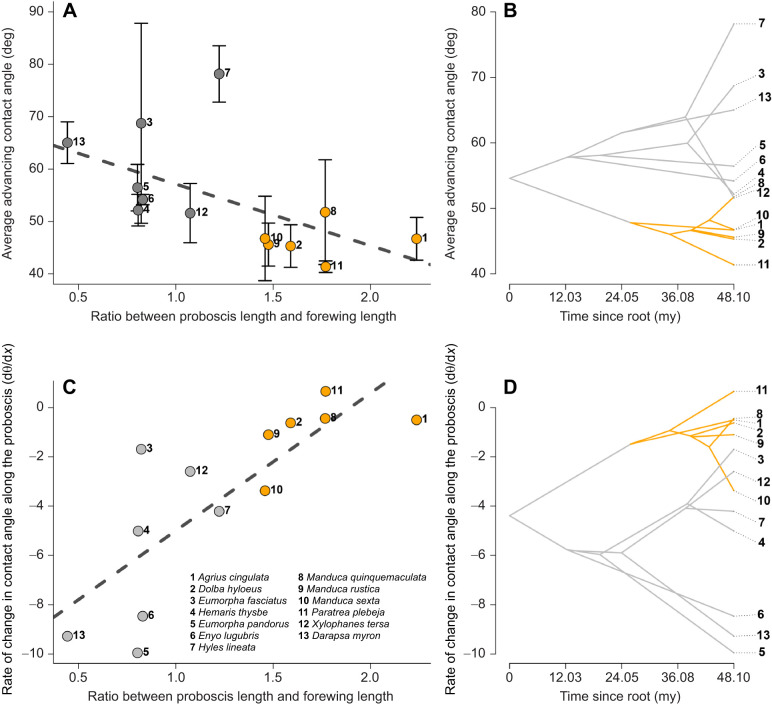
**As the proboscis becomes relatively longer, both the average advancing contact angle and the variation of contact angle along the proboscis decrease, and these patterns are phylogenetically clustered.** (A) Average contact angle and relative proboscis length are correlated. The dashed line denotes the phylogenetic regression line fitted with a Brownian motion evolutionary model and measurement error. Whiskers are standard deviations. (B) Phenogram showing members of the subfamily Sphinginae (orange) clustering with low values of contact angles, whereas members of the Macroglossinae (gray) show larger variation in average contact angle. (C) The change in advancing contact angle along the proboscis correlates with relative proboscis length. More negative values indicate that the advancing contact angle is decreasing at a faster rate, whereas values near zero indicate almost no change. (D) The pattern of variation is similar to the pattern in B. my, million years.

The evolutionary model that best fitted our data was Brownian motion for both the average advancing contact angle and the slope or gradient of the contact angle along the proboscis (Table S3b,c), suggesting that the traits were simulated as evolving without any preferences. Their evolution followed the random-walk strategy in the chosen space of physical and morphological characteristics. Relatively longer proboscises had lower average advancing contact angles (slope: −11.79 [95% CI: −21.19, −2.34]; *t*=−2.26; *P*=0.04; adjusted *R*^2^=0.25; Fig. 8A). Members of the subfamily Sphinginae formed a cluster with low advancing contact angles, whereas members of the subfamily Macroglossinae showed a higher variability in average contact angles (Fig. 8B). Members of the Macroglossinae, however, showed higher rates of change in advancing contact angles along the proboscis (Fig. 8C,D).

Relatively longer proboscises of species in the subfamily Sphinginae also showed a lower rate of contact angle change along the proboscis (slope: 5.58 [95% CI: 3.24, 7.92]; *t*=4.33, *P*=0.001, adjusted *R*^2^=0.59; Fig. 8C), suggesting almost the same chemical composition of the cuticle and surface roughness along the entire proboscis.

### Other Lepidoptera follow the butterfly, not the hawkmoth, motif of wettability

**Fig. 9.**
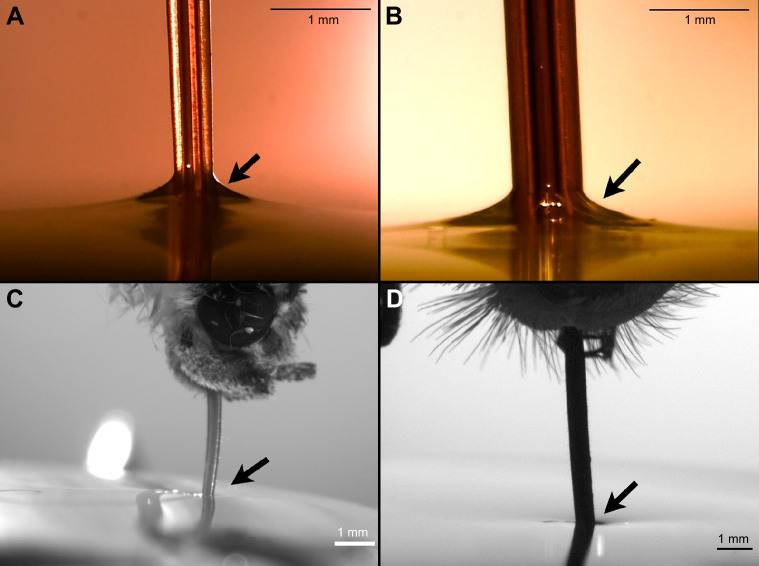
**Differences in the advancing contact angle of Lepidoptera.** (A,B) Meniscus formation (black arrow) between the proboscis and the water in *M. quinquemaculata* (A) and *M. rustica* (B). (C) Absence of a meniscus (black arrow) in the noctuoid moth *Catocala coccinata*, indicating the proboscis in this section is not hydrophilic. (D) Dimple formation between the proboscis and the water (black arrow) in the butterfly *Heliconius charithonia*, demonstrating that the proboscis is hydrophobic in this section.

We analyzed two other lepidopteran groups to understand whether wetting properties change depending on phylogenetic history or adult diet. First, we analyzed five species of a moth genus outside of hawkmoths, *Catocala* (superfamily Noctuoidea, family Erebidae), to understand whether phylogenetic history might influence wettability. None of the five species of *Catocala* showed hydrophilicity proximal to the tip region (Fig. 9), thus resembling the scenario of butterflies.

Second, we analyzed wetting properties of the butterfly *Heliconius charithonia* (Nymphalidae). The adult diet of *Heliconius* consists of nectar and pollen. Hydrophilicity, in this case, might be beneficial because it would allow pollen to be picked up easily by the proboscis. However, this species also showed a largely hydrophobic proboscis similar to that of other butterflies and the genus *Catocala* (Fig. 9D).

## DISCUSSION

We found that hawkmoths have hydrophilic proboscises. Probing the wetting properties of proboscises with water sets up the reference for nectar. Indeed, the surface tension of sucrose solutions changes little with increasing concentration; an increase of sucrose concentration up to 50% increases the surface tension of the solution to only about 5% (Kornev and Adler, 2019). However, sucrose or any sugar adds to the fluid stickiness, providing stronger adhesion to the surface. Therefore, hawkmoth proboscises cause fluids to spread over a larger area, in contrast to the more restricted hydrophilic proboscis tip of other Lepidoptera. The wetting properties of the proboscis are crucial for fluid uptake; a concentrated, thicker nectar will slow the uptake by the proboscis, but should not change the outcome (Salamatin et al., 2021).

The proboscis of hawkmoths also creates a large differential of capillary pressure for the liquid film to move from the external surface of the proboscis into the food canal (Salamatin et al., 2021; Zhang et al., 2018a). Thus, hawkmoths have a unique set of characteristics that enhance their ability to gather nectar passively. These proboscis features benefit hovering hawkmoths by ensuring the least amount of time spent in acquiring nectar, consequently increasing the rewards of drinking (Salamatin et al., 2021), offsetting energy expenditure, and reducing predation risks. These features might enable the proboscis to become longer over evolutionary time without compromising the ability for fluid uptake. No other investigated lineage of Lepidoptera has a similar pattern of wettability, not even pollen feeders (*Heliconius* butterflies) that would benefit from hydrophilicity.

### How a wettable proboscis facilitates drinking

When a hawkmoth dips the proboscis in a nectary, it can encounter nectar in different forms, from pools to droplets adhering to the walls (Pacini and Nepi, 2007). In parallel, hawkmoths have evolved a unique means to bring liquid into the food canal and transport it to the sucking pump. The hydrophilic proboscis of hawkmoths allows them to easily gather fluid regardless of the form in which it is encountered.

When nectar pools in the nectary, the proboscis of a hawkmoth is wetted over the entire area that contacts the nectar. Given that hawkmoths typically do not land on flowers to feed (Johnson et al., 2020), a large wettable surface allows them to gather as much nectar as possible in a single rapid dip. An additional line of evidence comes from the flowers visited by hawkmoths. Flower species that tend to be pollinated by hawkmoths typically produce large amounts of nectar that pools inside the floral tube, whereas flowers predominantly pollinated by butterflies produce less nectar (Pacini and Nepi, 2007). For instance, the long-tubed orchid, *Angraecum sororium* Schltr. 1925, has ∼6 cm of nectar pooled in its nectar spur (Wasserthal, 1997). The common pollinator of this orchid, the hawkmoth *Xanthopan morganii* (Walker 1856), has a proboscis that can reach up to 30 cm in length (22 cm on average; Arditti et al., 2012). If we assume the proboscis reaches the bottom of the nectary spur, with a single dip, *X. morganii* would wet 99–68% of the entire proboscis (see calculations in Supplementary Materials and Methods). If a hawkmoth needed to escape a predator after dipping its proboscis, it would leave with nectar adhering to the proboscis. If only the tip were wettable, the hawkmoth would need more time and energy hovering to acquire the pooled nectar.

Lepidoptera with long proboscises evolved different behavioral strategies to manage great viscous dissipation associated with liquid flow through the food canal (Tsai et al., 2014). Anti-parallel galeal sliding, cracking and bubble trains decrease viscous dissipation of energy. Galeal sliding has a twofold purpose. The first is to increase the food canal opening at the proboscis tip and, hence, to decrease the pressure differential required to move the liquid column (Tsai et al., 2014). The second is to enlarge the interlegular gaps so that liquid from the proboscis surface can flow through these gaps more freely. Proboscis cracking could be a more efficient strategy for long-tongued hawkmoths because the pressure differential generated by the sucking pump could be distributed over a shorter distance: head-to-crack rather than head-to-tip. Hence the driving pressure gradient increases. By making bubble trains, the insect reduces the friction of a continuous liquid column from the entire surface of the food canal to a significantly reduced surface area where the liquid bridges meet the food canal (Kornev and Adler, 2019).

Having a hydrophilic proboscis and large capillary pressure is crucial for hawkmoths with long proboscises. When nectar adheres to the walls of the nectary in droplets, the combination of wettability and morphology will enable the proboscis to collect the nectar as easily as if it were pooled. Hawkmoths can slide and twist their proboscises by muscles attaching at the base of the head, while also having fine control of the tip (e.g. for insertion into the nectary) by means of intrinsic muscles (Bänziger, 1970). Hence, hawkmoths can wipe the wall of the nectary to capture nectar droplets. The feeding performance of hawkmoths, therefore, might be unrelated to flower traits associated with how the nectar is produced, secreted or stored in the flowers.

### Wettability and capillary pressure as selection agents in proboscis elongation

Nectar feeding has evolved multiple times independently across insects, but hawkmoths have the longest proboscises relative to body size among all insects (Hutchinson et al., 2018; Reinwald et al., 2022). The co-evolution between proboscis length and floral nectary tubes is frequently evoked to explain why proboscises have elongated. Several studies dive into the type of selection that could elongate both the proboscis and the nectary tube (Hutchinson et al., 2018; Johnson et al., 2017; Sazatornil et al., 2016). However, no hypothesis explains the mechanism that allows proboscis elongation. In fact, several fluid mechanics models show that elongation of the proboscis should decrease drinking efficiency by requiring significant energy to pump fluid through it (Kingsolver and Daniel, 1979; Kornev et al., 2017; Tsai et al., 2014).

Our results are the first to shed light on the paradox of elongation, showing that hawkmoths with long proboscises take advantage of hydrophilicity to cover as much of the proboscis with nectar as possible, making the fluid film move into the food canal spontaneously, as theoretically suggested (Salamatin et al., 2021). The behavioral features of hawkmoth drinking suggest that their evolution has paralleled proboscis elongation to optimize drinking performance.

Having a highly hydrophilic proboscis with an almost constant small contact angle also allows hawkmoths to acquire more of the nectar that is displaced from the nectary when the insect dips its proboscis. Thus, nectar will also wet areas well beyond its pooling area as a result of the forced rise of the nectar column caused by the insertion of the proboscis. The capillary pressure differential will then ensure that the nectar will move toward the area where it can be absorbed.

When all these factors are combined, proboscis elongation will not necessarily be an issue for nectar absorption. The unique coupling of proboscis wetting properties and morphology might also be a key innovation that allowed hawkmoths to exploit a wider feeding niche while decreasing the physical constraints of proboscis elongation (Miller et al., 2023).

### Evolutionary covariation of proboscis length, wetting and capillary pressure

Most studies that evaluate feeding patterns show that species of hawkmoths and other Lepidoptera with long proboscises have a narrower niche when compared with species having shorter proboscises (Alarcón et al., 2008; Bauder and Karolyi, 2019; Johnson et al., 2017, 2020), a trend that we also observed. Although species with longer proboscises can feed from flowers with shorter tubes, they primarily feed from flowers with longer tubes (Alarcón et al., 2008; Bauder and Karolyi, 2019; Johnson et al., 2017, 2020; Moré et al., 2007). Our field observations, however, suggest that flowers with the shortest tubes are exploited only by hawkmoths with the shortest proboscises. Flowers with longer tubes represent a bigger reward (Johnson et al., 2017; Sazatornil et al., 2016; Wasserthal, 1997), but they also tend to be rarer and more ephemeral in most areas than are flowers with shorter tubes. If flowers with long tubes are rarer, individuals that feed on them would need to make the most of each feeding bout. Missing a feeding opportunity could incur an enormous fitness cost. Individuals with longer proboscises, such as members of the subfamily Sphinginae that can acquire nectar passively as a result of smaller contact angles, would have an advantage in these situations because they would acquire all nectar from these flowers more quickly. In such a scenario, the capillary pressure would be secondary to small contact angles.

Individuals with shorter proboscises, such as species of the Macroglossinae, would have a broader range of flower species from which they could feed (i.e. a larger niche breadth). Missing a feeding opportunity might not impact their fitness as much. If flowers are more abundant, the number of visits the moths make should be greater when compared with the number made by individuals that feed on flowers with longer nectar tubes. In such a scenario, individuals that can direct liquid faster to the absorption areas (i.e. legular bands) would have an advantage because they would be able to visit the next flower with the proboscis surface already dry. These two different feeding strategies, although leading to the same result (i.e. offsetting the metabolic demand of hovering), select for different morphological characteristics. However, the variation within Macroglossinae also suggests that some alternative strategies might be found in the group, warranting further investigation.

Our results suggest that phylogenetic structuring might be a prominent factor in explaining the observed trends. First, we found that adult dietary differences are not associated with a change in wetting properties of the proboscis. *Heliconius* is the only genus of Lepidoptera that routinely collects pollen, in addition to nectar, as a food source (Young and Montgomery, 2020). A hydrophilic proboscis as a high-energy surface would facilitate pollen capture. Angiosperm pollen grains are covered with a layer of lipid-rich material (Pacini and Hesse, 2005). Once the grains contact the hydrophilic surface of the proboscis, the oily layer tends to spread over the surface like oil over water. The high surface energy of a hydrophilic structure allows any type of a low surface energy material (e.g. proteins or lipids) to adhere more easily to the surface, minimizing the surface energy. Earlier observations (Kislev, 1972) indirectly confirm the proposed mechanism of nectar uptake by hawkmoths; pollen often accumulates along the dorsal and ventral legular bands on the hawkmoth proboscis, as it would be dragged by the flowing film.

This hydrophilicity of the proboscis surface explains why hawkmoths are frequently seen with pollen adhering to their proboscises (Kislev, 1972; Smith et al., 2022). But as we have shown, the proboscis of *H. charithonia* has the same hydrophobic–hydrophilic dichotomy as other butterflies. Dietary changes are thus unlikely to be associated with wettability of the proboscis. Second, we found that moths in a superfamily (Noctuoidea) closely related to the Sphingidae do not have an entirely wettable proboscis. The length of the proboscises of noctuoid moths is akin to that of butterfly proboscises, suggesting that length itself is not a driving force for changes in wettability. Both of these findings suggest that phylogenetic history might have played a major role in proboscis wetting patterns. Indeed, recent evolutionary models show that hawkmoth species that feed on similar flower species are more likely to show similar proboscis lengths than expected by phylogenetic history alone (Hutchinson et al., 2018). Therefore, changes in adult dietary preferences (i.e. changes in interaction partners) in the Sphinginae might have triggered changes in physical characteristics of the proboscis of species in this subfamily.

### Conclusion

We showed that the hawkmoth proboscis is consistently hydrophilic among all 13 species tested. This discovery positions hawkmoths as unique among all Lepidoptera that have been tested for wettability of the proboscis. For metrics of proboscis performance in passive collection of nectar, we used the advancing contact angle and the ratio *R*_o_/*R*_i_ of the radii of the galeal cross-section and food canal. Eqn 1 explains the importance of this ratio as the parameter characterizing the strength of the spontaneous pull of a nectar film from the proboscis surface into the food canal. These two characters are indicators of the unique ability of passive nectar collection by hawkmoths. The smaller the advancing contact angle, the larger the area of the proboscis that will be covered with a nectar film from a drop of the same volume. While a nectar film in the food canal is always pulling nectar from the external proboscis surface, the curvature of the proboscis aids in the capillary action. The larger the ratio *R*_o_/*R*_i_>1, the less important is the capillary push of the external film toward the legular band. The smaller the ratio *R*_o_/*R*_i_<1, the stronger the capillary push of the external film toward the legular bands. Thus, morphological features on the proboscis surface leading to *R*_o_/*R*_i_<1 facilitate fluid uptake.

Species with relatively longer proboscises have smaller contact angles. These proboscises are more wettable relative to the shorter proboscises of other species. But shorter proboscises have lower variation in the *R*_o_/*R*_i_ ratio. Thus, although longer proboscises can acquire nectar faster when dipped into a flower, shorter proboscises are fine tuned to direct the nectar to the absorption areas and hence into the food canal.

We showed that these two proboscis parameters (advancing contact angle and *R*_o_/*R*_i_) are reflected in the evolutionary history of hawkmoths and distinguish one hawkmoth subfamily, the Sphinginae, from another, the Macroglossinae. These two wetting characteristics might have enabled the proboscis to elongate to extreme sizes without decreasing feeding performance. We showed that other moths and butterflies do not have an entirely hydrophilic proboscis, suggesting that neither adult diet nor proboscis length necessarily triggers changes in wetting properties. We suggest that changes in wetting properties of the proboscis could be considered a key innovation that has significantly influenced the evolution of hawkmoths and allowed them to expand their feeding niche.

## Supplementary Material

10.1242/jexbio.245699_sup1 Supplementary information
