# Supplementary material for: Wettability and morphology of proboscises interweave with hawkmoth evolutionary history
Source: J Exp Biol. 2023 Oct 11;226(19):jeb245699. doi: 10.1242/jeb.245699 (PMC10617603; doi:10.1242/jeb.245699)
Supplement: Supplementary information [file jexbio-226-245699-s1.pdf]

# Supplementary Materials and Methods

## Contact angle measurements

Our first method of measurement stems from a previous custom-designed LabVIEW program used to measure contact angle of fibers (Sun et al., 2022; Zhan et al., 2022). For this code, we needed to specify the region of interest (green rectangle in Fig. S1a) to cover the menisci on either side of the proboscis at the water-air interface. The menisci are detected as seen in Fig. S1b. Simultaneously, the traced contours are plotted in Cartesian coordinates for the contact angle measurement, as shown in Fig. S1c. Then, the red "start" cursor $(x_{start}, y_{start})$ is selected where the meniscus meets the surface of the proboscis at the water-proboscis interface, and the blue "end" cursor $(x_{end}, y_{end})$ is used to specify the range of data for the parabola fitting. Depending on the magnification of the images, data points within 20 to 30 pixels in width ($20 \leq |x_{start} - x_{end}| \leq 30$) give the best parabola fitting, $y = ax^2 + bx + c$. Figure 2d shows the data as red squares and the black curve corresponding to the best parabola fitting. Thus, the contact angle can be given by $\theta = \pi - \tan^{-1}|y'(x_{start})|$, $y'(x_{start}) = 2ax_{start} + b$ is the slope where the meniscus meets the surface of the proboscis.

During our analyses of hawkmoth contact angles, we noticed that – because of the hydrophilicity of the hawkmoth proboscis – the videos were showing two contact angles: one on the lateral sides, and another on the legular bands of the proboscis. This made the measurements from the LabVIEW code less reliable. Because of that, we opted to also measure the contact angles using the angle tool in Fiji-ImageJ (Schindeling et al., 2012; Schneider et al., 2012). To

Journal of Experimental Biology • Supplementary information

ensure the measurements from the angle tool were repeatable, we measured both sides of the proboscis and average them. The measures made on both sides were highly consistent (Fig. S2) which ensured that the method was reliable. Further, to ensure our measures were robust, we compared the measurements taken from the LabVIEW code and the ImageJ measurements on a few select species. Given that the LabVIEW measures were slightly higher than the ones made with ImageJ, we performed a conservative correction to add the possible error measurement to our data. We divided the LabVIEW measure by the ImageJ measure to have a ratio of difference between these two methods, which showed that, on average, measures LabVIEW were 1.12 times larger than ImageJ measures (Fig. S3). We then multiplied the standard deviation of the final dataset by 1.12. Although that procedure increased the error in our data, it also ensured the reliability of our data. It is important to highlight that our results would not change regardless of the method used. All proboscises would still be entirely hydrophylic. Thus, the correction was solely to add a source of error to the data, keeping the data reliable.

Journal of Experimental Biology • Supplementary information

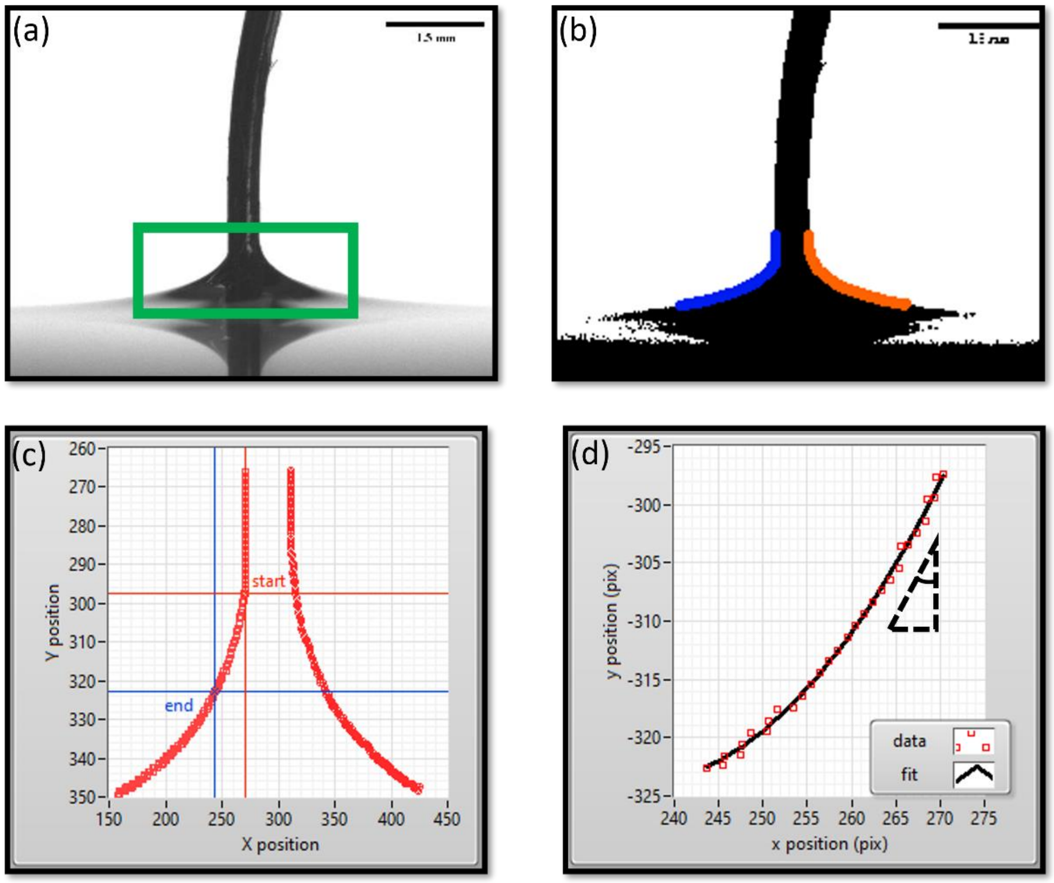


**Fig. S1.** Contact angle measurement using the designed LabView program. (a) Using the drawing tools to draw a rectangle around the menisci. (b) The contours of the menisci. (c) The contour of the menisci is traced using extracted coordinates. (d) At least 40 points are enclosed within the start and end line, which gives the slope.

Journal of Experimental Biology • Supplementary information

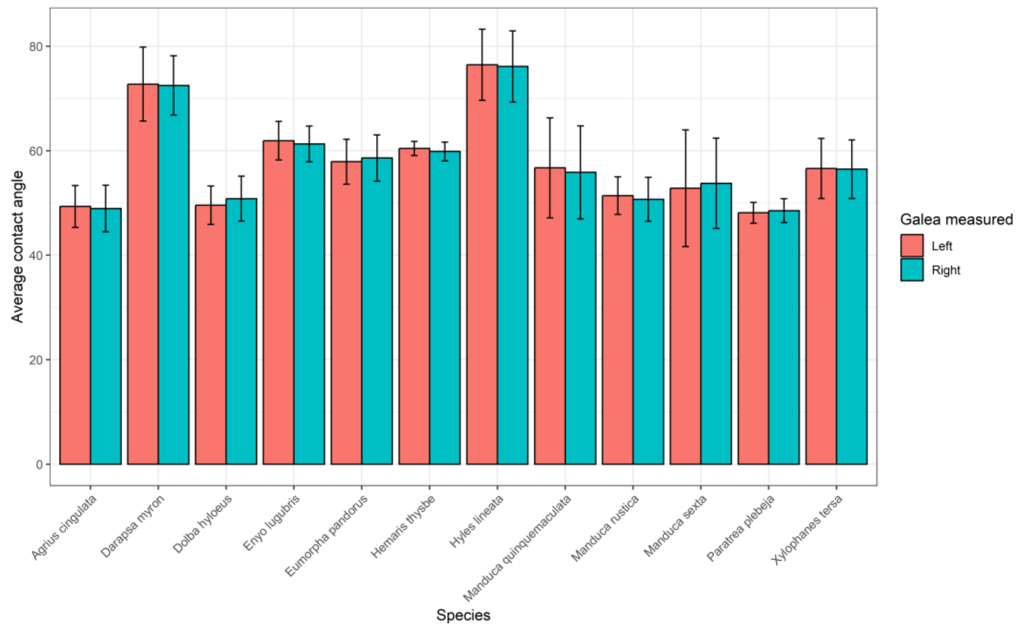


**Fig. S2**. Comparison of the measurements between the right and left side of the proboscis of the hawkmoths probed in our experiment. As can be seen, both sides had consistent measures.

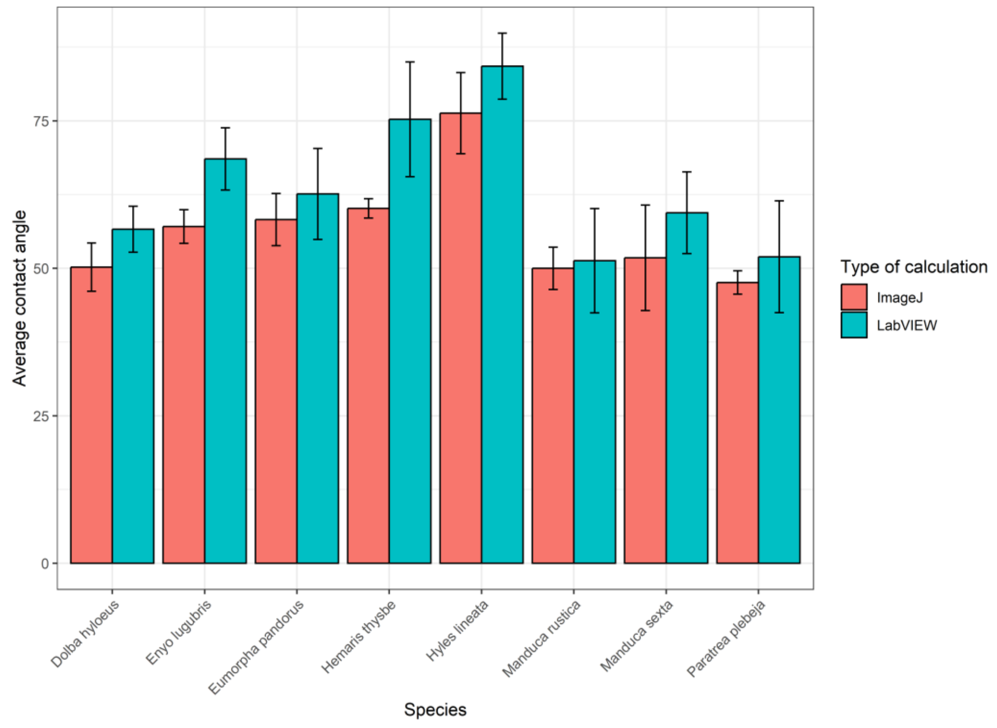


**Fig. S3.** Comparison between the methods to measure contact angle. LabVIEW showed consistently higher angles for the advancing contact angle. To account for this source of error, we multiplied the ratio between the averages of both measurements by the standard deviation in the main analysis.

Journal of Experimental Biology • Supplementary information

**Measuring the radius of curvature of the galea**

The ImageJ macro begins by having the user select nine points on the curvature of interest on the image of interest. Each three points is considered one set, where the last point of the first set is shared as the first point in the subsequent set. When all the points have been placed, there will be four sets of data.

Within a set, the code first calculates the distance between points by finding the side of a triangle. Then, by using the distances between the three points in a set, we can calculate the perimeter of the triangle. Using both the perimeter and the distances, we calculate the area of the circumcircle of the triangle using the following equation:

$$area = \sqrt{p * (p - d1) * (p - d2) * (p - d3)}$$

where $p$ is perimeter and $d$ is the distance of a given side of the triangle. Next, we calculate the circumradius using the following equation:

$$radius = \frac{(d1 * d2 * d3)}{\sqrt{((d1 + d2 + d3) * (d2 + d3 - d1) * (d3 + d1 - d2) * (d1 + d2 - d3)}}$$

To transform that measure from pixels to millimeters (or any other scale), we divide the measure by the ratio between pixels and millimeters obtained from the 'Set scale' function in ImageJ. To ensure the measurement is realistic, we found the centroids in $x$ and $y$ of the triangle and used the radius of the circumcircle to draw the circle. In a set, the dots are connected to make the triangle visible to the user.

Journal of Experimental Biology • Supplementary information

The process is then iterated to other sets of points. The process of placing the points of interest can change the metric being calculated. For instance, if the points are placed along various parts of a structure, it will measure how the radius of curvature changes along the curve of the structure of interest. If the points are placed in the same area multiple times, the same area will be measured four times, which can then be averaged. We placed the points of interest multiple times in the same structure and averaged the measurements of radius of curvature. Depending on the position of the cross-section of the proboscis, some sets did not provide realistic measures of the curvature. Thus, we only used the measurement if we could reliably average two sets of data. The code and a user manual will be uploaded as supplementary materials alongside the paper.

Journal of Experimental Biology • Supplementary information

**Table S1.** Average values (± standard deviation) of all the measurements taken from 13 species of hawkmoths (Lepidoptera: Sphingidae). Slope of change denotes the slopes of a relationship between average advancing contact angle and the section of the proboscis in which the angle was measured. Measurements of radii of curvature and tapering angle were made from CT-scans, thus representing the measurements taken from one individual per species.

| Species | Advancing contact angle, degrees | Contact angle hysteresis, degrees | Slope of change | Radius of curvature of the galea, mm | Radius of curvature of the food canal, mm | Tapering angle |
|---|---|---|---|---|---|---|
| *Manduca sexta* | 46.75 ± 7.15 | | -3.379 | 0.101 ± 0.03 | 0.062 ± 0.005 | 0.336 |
| *Manduca rustica* | 45.58 ± 3.63 | | -1.094 | 0.146 ± 0.02 | 0.085 ± 0.024 | 0.231 |
| *Manduca quinquemaculata* | 51.77 ± 8.85 | | -0.4343 | 0.128 ± 0.03 | 0.098 ± 0.007 | 0.226 |
| *Agrius cingulata* | 46.68 ± 3.63 | | -0.4952 | 0.123 ± 0.02 | 0.114 ± 0.004 | 0.113 |
| *Paratrea plebeja* | 41.35 ± 0.97 | | 0.6644 | 0.106 ± 0.07 | 0.045 ± 0.002 | 0.388 |
| *Dolba hyloeus* | 45.30 ± 3.61 | | -0.6163 | 0.074 ± 0.01 | 0.054 ± 0.001 | 0.297 |
| *Hyles lineata* | 78.17 ± 4.76 | | -4.215 | 0.094 ± 0.01 | 0.056 ± 0.012 | 0.411 |
| *Hemaris thysbe* | 54.18 ± 0.86 | | -8.462 | 0.077 ± 0.01 | 0.053 ± 0.020 | 0.464 |
| *Xylophanes tersa* | 51.58 ± 5.01 | | -2.596 | 0.109 ± 0.01 | 0.062 ± 0.014 | 0.281 |
| *Eumorpha pandorus* | 52.15 ± 2.67 | | -5.015 | 0.131 ± 0.03 | 0.077 ± 0.007 | 0.606 |
| *Eumorpha fasciatus* | 68.74 ± 16.92 | | -1.694 | 0.092 ± 0.09 | 0.067 ± 0.003 | 0.282 |
| *Darapsa myron* | 65.03 ± 3.53 | | -9.275 | 0.058 ± 0.03 | 0.042 ± 0.006 | 0.740 |
| *Enyo lugubris* | 56.45 ± 3.94 | | -9.95 | 0.138 ± 0.02 | 0.071 ± 0.003 | 0.300 |

**Table S2.** Average advancing contact angle with standard deviation along the proboscis of 13 species of hawkmoths. The normalized distance from the head denotes the region of the proboscis, with smaller values being closer to the head, while larger values are closer to the tip of the proboscis. These values were used to generate Figure 3 in the text.

| | Normalized distance from the head | | | | | | | |
|---|---|---|---|---|---|---|---|---|
| **Species** | **0.1-0.2** | **0.2-0.3** | **0.3-0.4** | **0.4-0.5** | **0.5-0.6** | **0.6-0.7** | **0.7-0.8** | **AVERAGE** |
| *Manduca quinquemaculata* | 54.8 ± 9.95 | 51.1 ± 9.28 | 50.2 ± 9.63 | 52.1 ± 9.88 | 50.5 ± 8.46 | 49.8 ± 8.29 | 56.6 ± 14.13 | 56.3 ± 9.24 |
| *Manduca rustica* | 49.5 ± 6.84 | 45.5 ± 5.16 | 45.1 ± 4.23 | 45.7 ± 4.05 | 43.1 ± 4.33 | 45.9 ± 3.44 | 44.1 ± 2.50 | 51.1 ± 3.88 |
| *Manduca sexta* | 56.6 ± 10.62 | 36.0 ± 18.94 | 47.4 ± 8.18 | 48.9 ± 9.31 | 45.0 ± 7.01 | - | - | 53.3 ± 9.90 |
| *Agrius cingulata* | 48.8 ± 2.92 | 47.6 ± 5.54 | 47.3 ± 4.15 | 46.3 ± 4.34 | 45.6 ± 4.65 | 45.8 ± 3.98 | 45.4 ± 2.83 | 49.1 ± 4.22 |
| *Paratrea plebeja* | 43.1 ± 2.05 | 40.6 ± 2.73 | 40.9 ± 1.98 | 40.1 ± 3.66 | 41.2 ± 2.61 | 42.3 ± 1.97 | - | 48.3 ± 2.14 |
| *Dolba hyloeus* | 48.2 ± 5.83 | 44.2 ± 5.49 | 44.2 ± 5.45 | 44.9 ± 3.52 | 43.8 ± 4.54 | 45.0 ± 3.88 | 46.6 ± 3.53 | 50.2 ± 3.99 |
| *Hyles lineata* | 90.0 ± 0.00 | 84.7 ± 8.44 | 67.0 ± 12.35 | 77.1 ± 12.97 | 69.6 ± 9.45 | 76.4 ± 9.69 | 55.3 ± 1.43 | 76.2 ± 6.79 |
| *Enyo lugubris* | 86.0 ± 8.00 | 59.0 ± 6.68 | 46.6 ± 3.76 | 44.5 ± 1.62 | 43.5 ± 1.24 | - | - | 61.6 ± 3.55 |
| *Eumorpha pandorus* | 68.0 ± 13.15 | 51.7 ± 5.11 | 48.0 ± 2.97 | 46.0 ± 2.29 | 45.8 ± 2.63 | - | - | 58.2 ± 4.36 |
| *Eumorpha fasciatus* | 60.5 ± 2.12 | 52.5 ± 0.70 | 47.0 ± 1.41 | 48.5 ± 0.70 | 52.5 ± 2.12 | 53.5 ± 0.70 | 50.5 ± 2.12 | 51.8 ± 3.92 |
| *Hemaris thysbe* | 90.0 ± 0.00 | 48.3 ± 3.83 | 45.2 ± 1.35 | 44.7 ± 1.38 | 42.7 ± 2.87 | - | - | 60.1 ± 1.57 |
| *Xylophanes tersa* | 63.7 ± 13.64 | 52.9 ± 5.42 | 50.5 ± 5.71 | 49.8 ± 5.84 | 49.6 ± 5.98 | 47.7 ± 4.61 | 48.0 ± 4.21 | 56.6 ± 5.68 |
| *Darapsa myron* | 90.0 ± 0.00 | 78.2 ± 11.75 | 65.7 ± 9.75 | 58.0 ± 9.00 | 53.7 ± 7.75 | - | - | 72.6 ± 6.37 |

Journal of Experimental Biology • Supplementary information

**Table S3.** Akaike Information Criterion values of the evolutionary models fitted to the phylogenetic linear regression models. Models with ΔAIC ≥ 2 were considered poorly fit. When competing models had ΔAIC ≤ 2, we chose the model with the fewest parameters.

| | AIC | ΔAIC |
|---|---|---|
| *(a) Proboscis curvature* | | |
| Brownian motion | 14.519 | - |
| Lambda | 14.519 | 0 |
| Delta | 16.497 | 1.977 |
| Kappa | 16.519 | 2 |
| *(b) Tapering angle* | | |
| Brownian motion | -4.355 | - |
| Lambda | -4.355 | 0 |
| Delta | -2.355 | 2 |
| Kappa | -2.355 | 2 |
| *(c) Average advancing contact angle* | | |
| Brownian motion | 100.263 | - |
| Lambda | 100.263 | 0 |
| Kappa | 102.263 | 2 |
| Delta | 102.263 | 2 |
| *(d) Slope of change in advancing contact angle* | | |
| Brownian motion | 64.254 | - |
| Lambda | 64.254 | 0 |
| Delta | 65.436 | 1.182 |
| Kappa | 66.254 | 2 |

**Calculation of proboscis dipped volume in *Xanthopan morgani***

To calculate how much the nectar pooled in the orchid *Angraecum sororium* would rise when the proboscis is dipped, we used data published in Wasserthal (1997), Arditti et al. (2012), and an image of *A. sororium* taken from the Herbarium Jany Renz. From Wasserthal (1997), we extracted the volume of nectar in the flower and the range of proboscis lengths. From Arditti et al. (2012), we used the image of *X. morgani* with the proboscis uncoiled to measure

Journal of Experimental Biology • Supplementary information

the width of the proboscis along its entire length. For the image of *A. sororium*, we measured the width and length of the nectar spur of the species. Given the conservation of volume, the nectar volume displaced by the proboscis after it is dipped in the spur should be equal to the volume of the proboscis submersed in the nectar. Modeling the spur lumen and proboscis as straight cones and knowing from Wasserthal (1997) the height $h$ of the nectar cone at its highest level in the spur before submersing the proboscis in it, and using the radius $r_p$ of the proboscis at distance $h$ from its tip, we calculate the volume ($V = (1/3)\pi r_p^2 h$) of nectar displaced by proboscis.

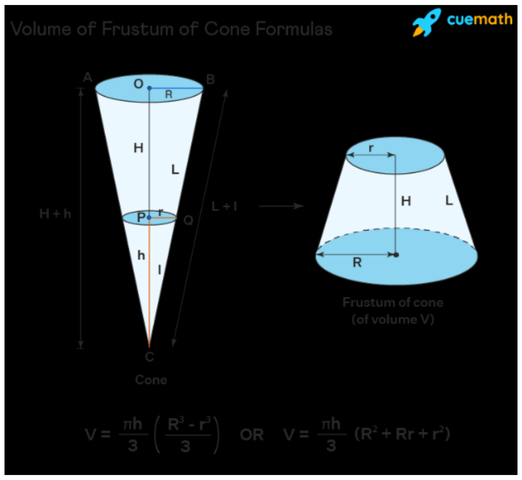


This volume goes into the gap formed by the submersed proboscis and the spur lumen. To find the new height $h + H$, we subtract the volume of the proboscis cone at new height, $V_p = (1/3)\pi(r_p/h)^2(h + H)^3$, from the volume of the spur cone, $V_{sp} = (1/3)\pi(r_{sp}/h)^2(h + H)^3$. at the same height, $h + H$ , where $r_{sp}$ is the radius of the spur lumen at height $h$ from the bottom. This difference must be equal to the displaced volume,

$$\left(\frac{1}{3}\right)\pi {r_{sp}}^2 h = \left(\frac{1}{3}\right)\pi(r_{sp}/h)^2(h + H)^3 - \left(\frac{1}{3}\right)\pi(r_p/h)^2(h + H)^3$$

Solving this equation for wet length of the proboscis, $h + H$, we obtain:

$$h + H = h\sqrt[3]{{r_{sp}}^2/(r_{sp}^2 - {r_p}^2)}$$

Journal of Experimental Biology • Supplementary information

Values used and obtained are below.

Mean spur radius ($r_{sp}$) = 0.0709 cm

Mean proboscis radius ($r_p$) = 0.089 cm

Nectar height ($h$) = 17.3 cm

New nectar height ($h + H$) = 20.43 cm

Proboscis length ($L$) = from 20.5 to 30 cm

Proboscis covered in nectar (%) = from 99.43 to 68.12

Journal of Experimental Biology • Supplementary information

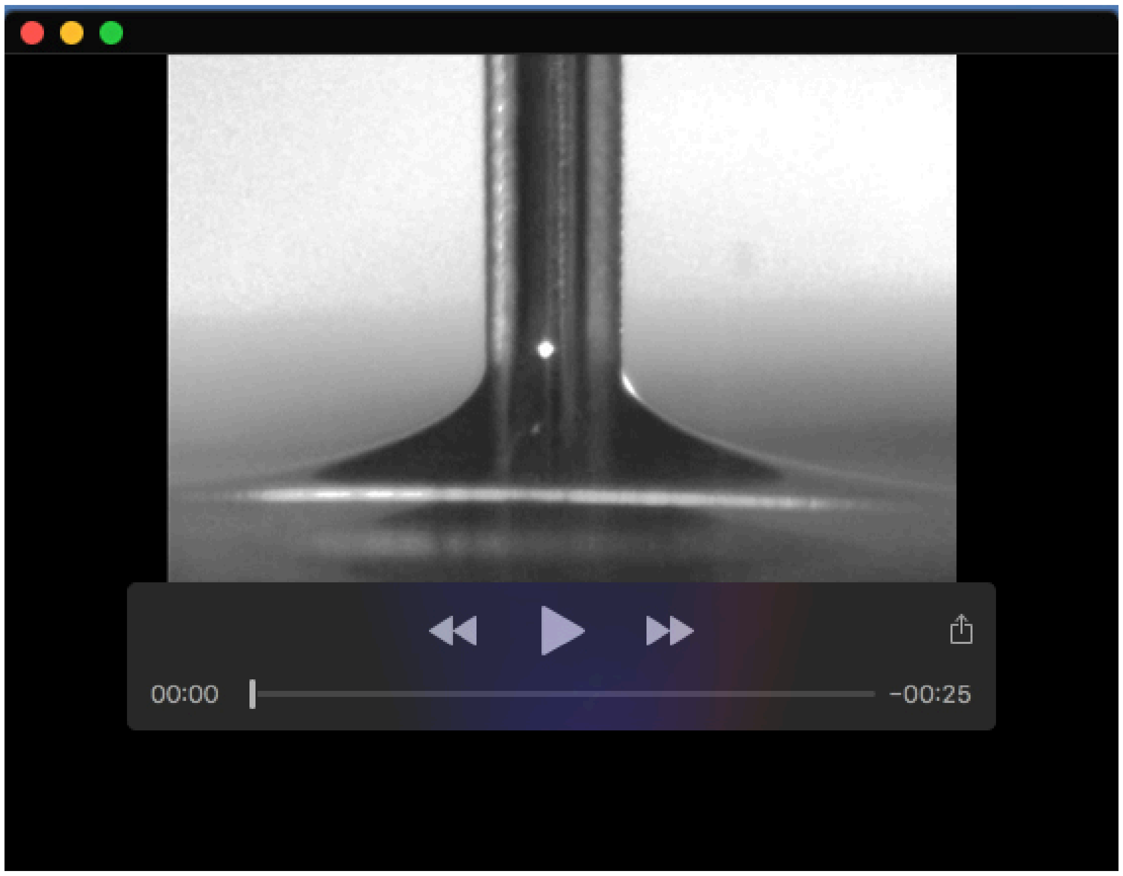


**Movie 1.** Movie showing how the galeae can crack and open the food canal during drinking. The species in the video is *Manduca rustica*. See Figure 5 in the main manuscript for an schematic of how the cracking works.

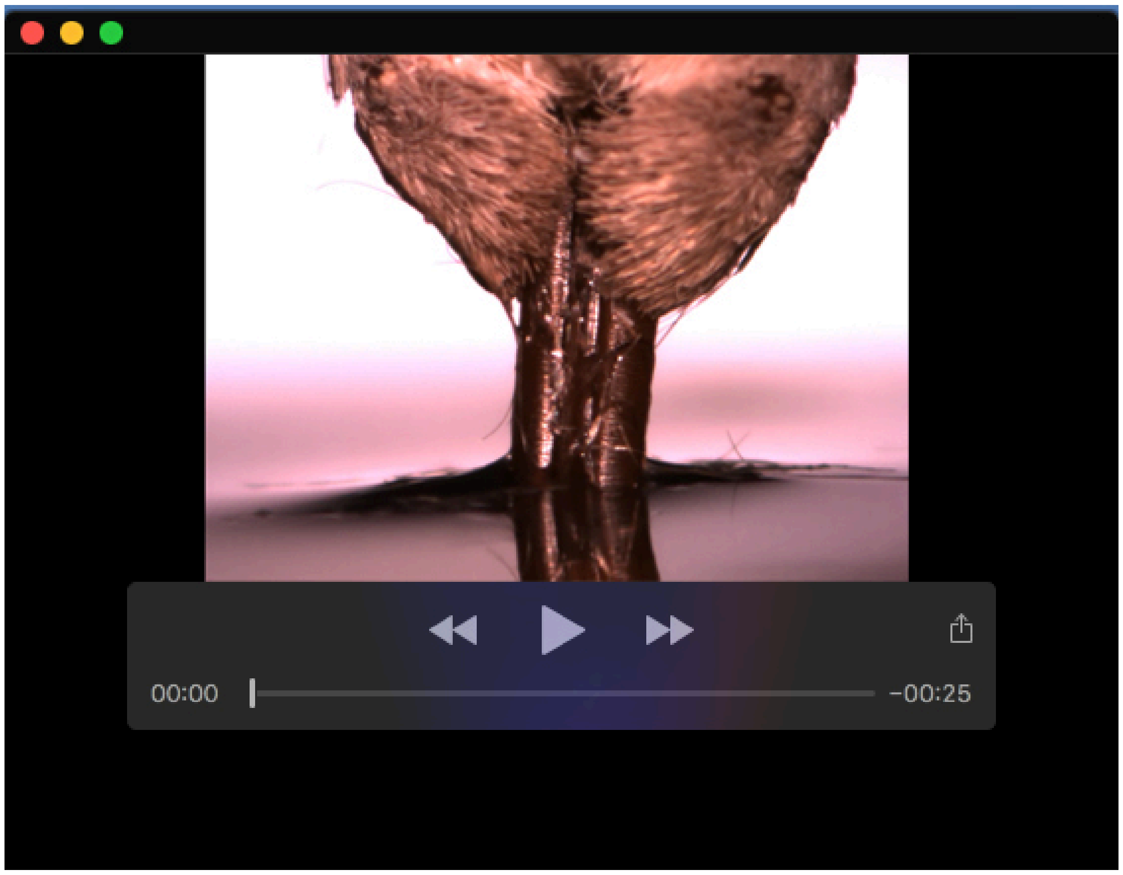


**Movie 2.** Anti-parallel sliding of the galea in *Eumorpha pandorus*. The galeae move in opposite directions during drinking, which increases the spaces between the legulae and can open tip of the proboscis.

Journal of Experimental Biology • Supplementary information

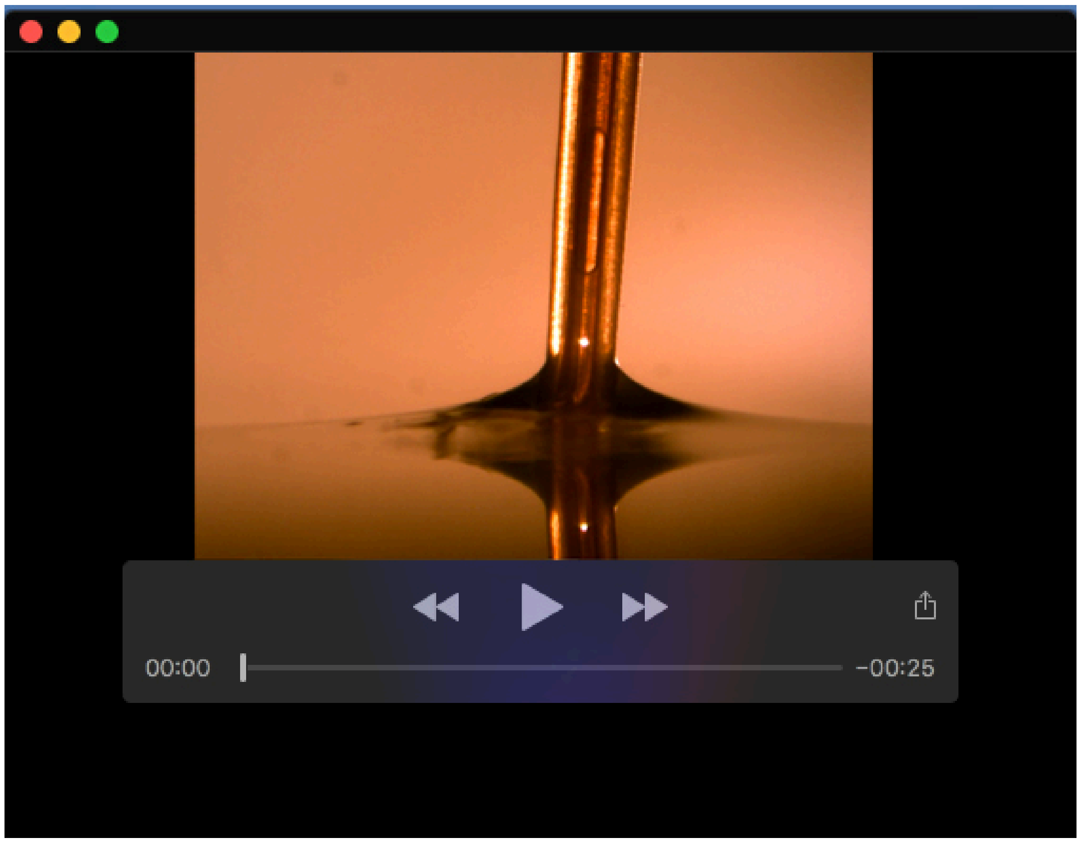


**Movie 3.** Bubble train inside the food canal being pushed towards the head in *Manduca quinquemaculata*.

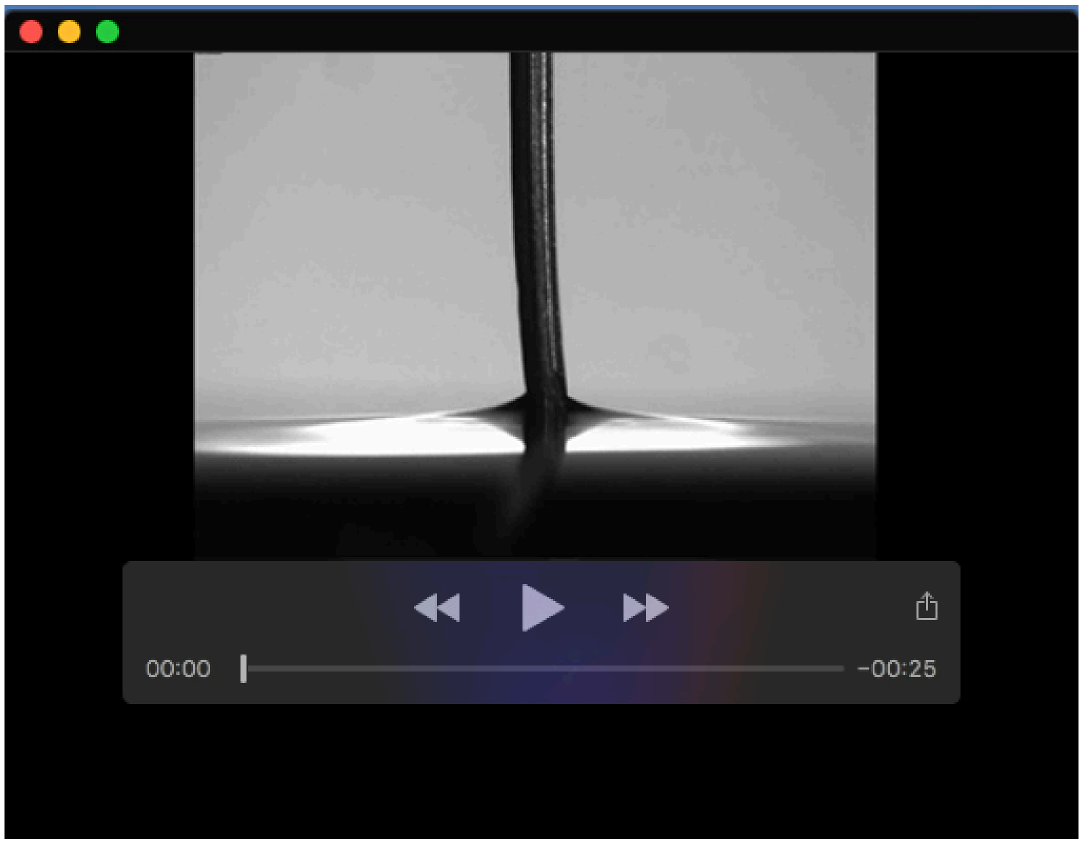


**Movie 4.** Capillary experiment performed with a single galea of a dead hawkmoth. The galea is positioned at an angle to the camera, showing simultaneously the food canal and the outside of the proboscis. The single galea is lowered towards DI water, and the water rushes in the food canal as soon as the galea touches the water. Meanwhile, the contact angle on the external surface of the proboscis is lower and the water does not rise there.

Journal of Experimental Biology • Supplementary information

**REFERENCES**

Arditti, J., Elliott, J., Kitching, I. J., & Wasserthal, L. T. (2012). 'Good Heavens what insect can suck it'–Charles Darwin, *Angraecum sesquipedale* and *Xanthopan morganii praedicta*. *Botanical Journal of the Linnean Society*, 169(3), 403-432.

Schindelin, J., Arganda-Carreras, I., Frise, E., Kaynig, V., Longair, M., Pietzsch, T., … Cardona, A. (2012). Fiji: an open-source platform for biological-image analysis. *Nature Methods*, *9*(7), 676–682.

Schneider, C. A., Rasband, W. S., & Eliceiri, K. W. (2012). NIH Image to ImageJ: 25 years of image analysis. *Nature Methods*, *9*(7), 671-675.

Sun, Y., Ma, J., Peng, F., & Kornev, K. G. (2022). Making droplets from highly viscous liquids by pushing a wire through a tube. *Physics of Fluids*, 34(3), 032119.

Wasserthal, L. T. (1997). The pollinators of the Malagasy star orchids *Angraecum sesquipedale*, *A. sororium* and *A. compactum* and the evolution of extremely long spurs by pollinator shift. *Botanica Acta*, 110(5), 343-359.

Zhang, Z., Peng, F., & Kornev, K. G. (2022). The Thickness and Structure of Dip-Coated Polymer Films in the Liquid and Solid States. *Micromachines*, 13(7), 982.

Journal of Experimental Biology • Supplementary information
